# Bovine Leukemia Virus and Human Breast Cancer: A Review of Clinical and Molecular Evidence

**DOI:** 10.3390/v17030324

**Published:** 2025-02-26

**Authors:** Rancés Blanco, Claudio Quezada-Romegialli, Juan P. Muñoz

**Affiliations:** 1 Independent Researcher, Av. Vicuña Mackenna Poniente 6315, La Florida 8240000, Chile; rancesblanco1976@gmail.com; 2 Plataforma de Monitoreo Genómico y Ambiental, Departamento de Química, Facultad de Ciencias, Universidad de Tarapacá, Arica 1000007, Chile; clquezada@academicos.uta.cl; 3 Laboratorio de Bioquímica, Departamento de Química, Facultad de Ciencias, Universidad de Tarapacá, Arica 1000007, Chile

**Keywords:** breast cancer, bovine leukemia virus, zoonotic, human infection, viral oncogenesis

## Abstract

Despite significant advancements in early diagnosis and treatment, breast cancer (BC) remains a major global health challenge. Ongoing research is essential to identify novel risk factors, implement innovative screening programs, and develop personalized treatment approaches. Among the various risk factors, infection with certain oncogenic viruses has emerged as a potential contributor to BC development. Increasing evidence suggests that bovine leukemia virus (BLV) may contribute to zoonotic infections in humans, with a potential role in BC initiation and progression. This review evaluates clinical and experimental data on BLV presence in both malignant and non-malignant breast tissues, exploring potential mechanisms through which BLV may access human breast tissue and contribute to carcinogenesis. Current data reveal a higher prevalence of BLV infection in BC tissues compared to non-tumor tissues, correlating with an increased risk of BC development. In this context, dairy and meat products from BLV-infected animals have been proposed as potential transmission sources. BLV-encoded proteins disrupt key oncogenic pathways, which support their possible role in breast carcinogenesis. However, the interpretation of these findings is limited by potential confounding factors such as genetic predisposition, environmental exposures, and dietary influences. Further research, including well-controlled epidemiological studies, longitudinal cohorts, and mechanistic investigations into BLV proteins in human breast cells, is necessary to determine its role in BC development.

## 1. Introduction

Breast cancer (BC) is the most commonly diagnosed malignancy among women globally and the leading cause of cancer-related death in this population [1]. In the United States alone, an estimated 310,720 new cases and 42,250 deaths from BC among women were reported for 2024 [2]. Notably, the highest BC incidence rates occur in regions such as Australia/New Zealand, Northern America, Northern Europe, and Western Europe, where the rates are nearly three times higher compared to those in Eastern Africa, South-Central Asia, and Middle Africa [1,3]. In general, the differences in BC incidence rates among countries and ethnicities are associated with differences in the exposure level to risk factors and the implementation and/or access to primary prevention programs [3].

A variety of reproductive and lifestyle risk factors have been associated with BC development, including, but not limited to, age at menarche and menopause, number of children, breastfeeding, use of hormonal therapy, obesity, genetic mutations, family history, and unhealthy food habits [4]. Furthermore, the potential relation of some viruses like mouse mammary tumor virus (MMTV), Epstein-Barr virus (EBV), human papillomavirus (HPV), and human cytomegalovirus (HCMV) to BC development has also been proposed [5,6,7,8,9]. In addition, a potential association between viral co-infections and BC development was previously suggested [10]. However, it is well accepted that breast carcinogenesis is most related to a combination of environmental, genetic, and lifestyle risk factors [11].

Particularly, the potential association of BC development and the intake of animal products have been addressed in numerous studies [12,13,14]. For instance, bovine meat and dairy products could contain high levels of saturated fats [15,16], xenoestrogens, growth factors [17], and endocrine disruptors such as heterocyclic amines found in processed or cooked red meat [18,19], all of which may contribute to BC development and progression [20]. Despite these findings, the causal relationship between dietary factors and BC remains unclear.

Interestingly, the presence of the bovine leukemia virus (BLV) DNA in bovine milk and meat for human consumption has been reported [21,22,23]. BLV is a deltaretrovirus that naturally infects cattle, zebu, and buffalo [24,25]. BLV is the causative agent of enzootic bovine leucosis (EBL), characterized by persistent B-cell lymphocytosis and lymphoma development [26]. In addition, BLV is able to infect a broad variety of cells, including bovine mammary epithelial cells [27,28]. Recently, a meta-analysis conducted by Bushi et al. (2024), including 48 studies and 101,120 cattle samples, reported a global prevalence of BLV at 26.8% (95% CI: 20.0–33.0) [29].

The occurrence of anti-BLV antibodies was demonstrated in human females [21,30,31], which supports previous exposure to the virus. Moreover, BLV DNA was detected in blood samples from human females [30,32], which was also genetically related to the virus-infected cattle in the same geographical area and time period [33]. Furthermore, an association between the increased consumption of dairy products by females and the risk of BLV infection was reported [34], suggesting that bovine products could be a route for zoonotic BLV infection in humans.

Given the high consumption of dairy products in certain regions, BLV exposure may have public health implications. While its role in human disease remains uncertain, potential transmission through dairy consumption warrants further investigation to assess associated risks and preventive measures. In this regard, the presence of BLV has been frequently detected in breast tissue samples, and it has also been related to an increased risk of BC development [35,36,37]. Notably, the presence of BLV was demonstrated in initial benign breast tissues and in the posterior BC specimens from the same patients [38]. However, the relationship between BLV and BC remains highly controversial due to inconsistencies in the available data. While some studies report a strong association, others fail to find significant differences in BLV prevalence between BC cases and controls. These discrepancies may be attributed to variations in detection methodologies, sample sizes, and study designs. Differences in BLV DNA target regions, geographic variations in viral strains, and potential confounding factors further complicate the interpretation of findings.

This review provides a comprehensive analysis of the current clinical evidence linking BLV infection to human BC development. Additionally, it explores the potential mechanisms by which BLV enters breast epithelial cells and examines its contribution to BC initiation and progression.

## 2. BLV Structure and Lifecycle

**Figure 1:**
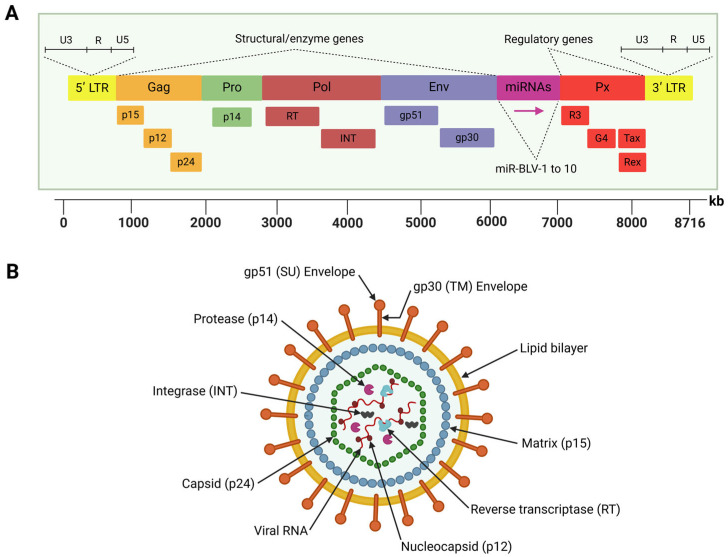
Schematic representation of the BLV genome structure (**A**) and viral particle (**B**). (**A**) The BLV genome encodes structural proteins and viral enzymes, regulatory proteins, and accessory proteins. It is flanked by two identical long terminal repeat (LTR) sequences. The region located between the *env* and *R3* genes encodes ten viral microRNAs (miRNAs). (**B**) The BLV particle has a spherical shape with the lipid bilayer envelope surrounding the icosahedral nucleocapsid that contains the viral genome inside. Created by BioRender.com.

BLV is a diploid single-stranded RNA that belongs to the Retroviridae family and *Deltaretrovirus* genus [39]. This virus is closely related to the human T-cell leukemia virus type 1 (HTLV-1), both of which are characterized by their ability to infect lymphocytes and integrate their genetic material into the host genome—a process known as establishing the proviral state—which is an essential hallmark of retroviral replication [40]. The BLV genome comprises an 8714-nucleotide sequence, enclosed by an icosahedral nucleocapsid surrounded by a double-layered lipid membrane structure derived from the host cell during viral budding [41]. This genome encodes structural proteins, viral enzymes, regulatory proteins, and accessory proteins. It is flanked by two identical long terminal repeat (LTR) sequences, the 5′ LTR and 3′ LTR, which regulate viral gene expression and replication [42] (Figure 1).

The 5′ LTR contains promoter elements that drive the activity of RNA polymerase II (RNAPII), which transcribes BLV protein-coding genes. These include the structural genes (*gag*, *pro*, *pol*, and *env*), regulatory genes (*tax* and *rex*), and accessory genes (*R3* and *G4*), all of which are essential for the virus lifecycle [43].

The *gag* gene encodes three non-glycosylated and structural proteins: the nucleocapsid protein (p12), the matrix protein (p15), and the other nucleocapsid protein (p24), which form the viral core [44]. The *pro* gene encodes the viral protease (p14), which cleaves gag and gag-pol polyproteins during virion maturation, a process critical for the release of infectious particles [45]. However, the *pol* gene encodes two essential enzymes: reverse transcriptase (RT), which converts viral RNA into double-stranded proviral DNA, and integrase (INT), which facilitates the integration of this DNA into the host genome [46]. The *env* gene encodes two glycosylated envelope proteins, glycoprotein (gp)51 (surface) and gp30 (transmembrane). The gp51 protein mediates virus–host cell fusion, while gp30 is involved in signal transduction pathways and stabilizing gp51 [45]. Notably, the *env* gene exhibits genetic polymorphism, with eleven distinct BLV genotypes identified through gp51 sequencing and phylogenetic studies. These genotypes are distributed differently across the globe, reflecting the evolutionary adaptation of BLV and geographical spread [47,48,49,50,51].

Located between the *env* gene and the 3′ LTR, the pX region encodes key regulatory proteins (Tax and Rex) and accessory proteins (R3 and G4) [52]. Tax functions as a transcriptional activator, stimulating LTR activity and driving viral gene expression while playing a critical role in BLV-induced tumorigenesis and lymphoproliferative disorders [53,54,55]. Likewise, Rex is involved in post-transcriptional regulation, ensuring the efficient export of unspliced viral RNA from the nucleus and supporting the synthesis of structural proteins [56]. Meanwhile, the accessory proteins R3 and G4 play critical roles in maintaining high viral loads during persistent infections, with G4 also associated with oncogenic activity, further highlighting its role in BLV pathogenesis [57,58].

Another unique feature of the BLV genome is a region located between the *env* and *R3* genes, which encode ten viral microRNAs (miRNAs) under the control of RNA polymerase III [59]. These miRNAs are involved in various biological processes, including immune modulation, cell signaling, apoptosis, and tumorigenesis [60]. These small RNA molecules contribute to immune evasion and viral persistence by downregulating host antiviral responses.

Some of BLV-encoded miRNAs share sequence identity with human miRNAs, suggesting a potential overlap in the target molecules of both viral and human miRNAs. For instance, it was found that miR-B2-5p displays a common seed overlap with human miR-943 [61]. The overexpression of miR-943 was evidenced in the stem population of primary human mammalian epithelial cells (HMECs), and it was also upregulated in cells when the tumor suppressor p53 was knocked down [62]. Moreover, miR-943 is negatively correlated with the gene expression of Ataxia-telangiectasia mutated (ATM) and breast cancer gene 1 (BRCA1) in BC, which suggests its potential involvement in the repair of DNA double-strand breaks [63]. Furthermore, it was found that BLV-miR-B4–3p also has a matching seed sequence with the human miR-29a [61]. The miR-29a targets SUV420H2 and downregulates the trimethylation of the histone H4K20, inducing the epithelial-to-mesenchymal transition (EMT), migration, and invasion of BC cells [64]. These facts together may suggest a possible oncogenic role of BLV miRNAs in human breast cells.

In the BLV lifecycle, a defining step is the integration of the virus as a provirus into the genomic DNA of peripheral blood cells, establishing a persistent infection in the host [65]. The retroviral lifecycle can be divided into two phases: the early phase and the late phase. The early phase includes viral entry, reverse transcription, and proviral integration. Viral envelope proteins mediate entry by interacting with specific host cell receptors. Inside the cell, viral RNA is reverse transcribed into DNA, which is then integrated into the host genome by the viral integrase enzyme, while the late phase involves the transcription and translation of viral RNA, assembly of new virions, and their release through budding [66].

Both phases require coordinated interplay between viral and host factors [67,68]. For example, the processing of viral proteins relies not only on the viral protease but also on host proteases, highlighting the viral dependence on host cellular machinery [69]. Efficient transcription of the BLV proviral genome is driven by the viral transcriptional activator Tax, which works in tandem with host factors to regulate viral gene expression [70]. Additionally, the LTR interacts with host transcription factors to fine-tune viral replication, demonstrating the intricate relationship between the virus and its host. This complex lifecycle and genetic makeup allow BLV to evade immune responses, persist in host populations, and contribute to oncogenesis, making it a significant concern for animal health and a useful model for studying retroviral biology.

BLV can also infect and replicate in cells from non-bovine animal species (e.g., lamb, canine, feline, murine, and human cells) [70,71]. However, the ability of BLV to infect non-bovine cells depends on diverse factors, such as the competence and response of the host immune system, the presence of viral receptors on the cell surface, and the capacity of BLV to hijack and customize the host cell machinery for replication [27]. For example, the expression of BLV precursor and mature proteins was also reported in COS-1 cells (African green monkey kidney cells) and FLK cells (fetal lamb kidney cells) transfected with wild-type proviral DNA [71]. Notably, it was demonstrated that the expression of gPr72Env, Pr70Gag, and pr45Gag precursor proteins as well as gp51, gp30, and p24 mature proteins was detected in the lysates of 293T human cells transfected with an infectious BLV clone. Moreover, the production and secretion of BLV viral particles by 293T-transfected cells was verified [72]. These facts together may suggest similarities in the BLV lifecycle across cells from different animal species, including human cells.

## 3. Routes for BLV Infection in Human Breast Tissues

The BLV is an enzootic retrovirus that naturally infects cattle, zebu, and buffalo [24,25], causing B-cell lymphomas in 1–5% of infected cattle [73]. In addition, BLV can be experimentally transmitted to other species, including goats [74], sheep [75], chickens [76], rabbits [77], and rats [78], which provide evidence concerning the capacity of BLV to cross species barriers. Remarkably, in humans, the presence of BLV has been detected in breast [79] and lung [80] tissues, as well as in blood cells [30].

In cattle, BLV is detected in biological fluids such as blood, colostrum, and milk, which are considered potential sources for viral transmission [81,82]. BLV can be transmitted horizontally through iatrogenic procedures permitting blood or fluid transfer like rectal palpation using contaminated sleeves, injections with reused needles, or animal-to-animal contact through nasal excretions or saliva [83]. Vertical transmission occurs from BLV-infected dams to calves mainly through the consumption of infected colostrum or milk [84].

BLV DNA was detected in 38.9% of the milk samples and in 32.1% of the meat samples obtained from BLV-positive cows [21]. Similarly, the BLV proviral *gag* segment was found in 48.0% of the milk samples and in 50.0% of the meat samples for human consumption [22]. Moreover, the ex vivo infectivity of milk cells from BLV-infected cattle was previously demonstrated [85], which suggests that milk and meat consumption could be a route for BLV infection in humans.

On the other hand, BLV DNA was detected in 33/95 (34.7%) samples of leukocytes and platelets (buffy coat cells) from the blood from human females using PCR and DNA sequencing [30]. Similarly, BLV DNA was found in 16.5% (33/200) of blood samples from women without BC [32]. Interestingly, Canova et al. reported a genetic relationship between the BLV DNA sequences found in the blood from virus-infected cattle and in women’s breast tissue in the same geographical area and time period [33].

Additionally, Buehring et al. detected antibodies against BLV capsid protein (p24) in 191/257 (74.3%) samples of the analyzed human sera. The distribution for the three antibody isotypes tested was as follows: IgG = 101/257 (39.3%), IgM = 80/257 (31.1%), and IgA = 96/256 (37.5%) [31]. However, the same authors reported no significant correlation between the occurrence of anti-BLV p24 antibodies and the presence of BLV DNA in the blood samples [30]. However, de Quadros et al. reported the presence of anti-BLV antibodies in 4.1% of the tested human serum samples [21]. The presence of anti-BLV antibodies in human sera provides evidence of previous exposure to the virus, although others have reported the lack of BLV antibodies in human serum samples [86].

Overall, the evidence suggests that uncooked or partially cooked meat and unpasteurized milk derived from BLV-infected cattle could be a potential source of a zoonotic infection to humans. In this regard, a statistically significant association was reported between an increased consumption of dairy products by females and the risk of BLV infection (OR = 2.4, CI 95%: 1.063–5.527; *p* = 0.035), although no relation with meat consumption was obtained in this study [34]. Remarkably, it was suggested that another oncogenic virus in humans (high-risk HPV) could reach the breast tissue through circulation (blood or lymphatic systems) in patients with HPV-positive cervical cancer [87].

**Figure 2:**
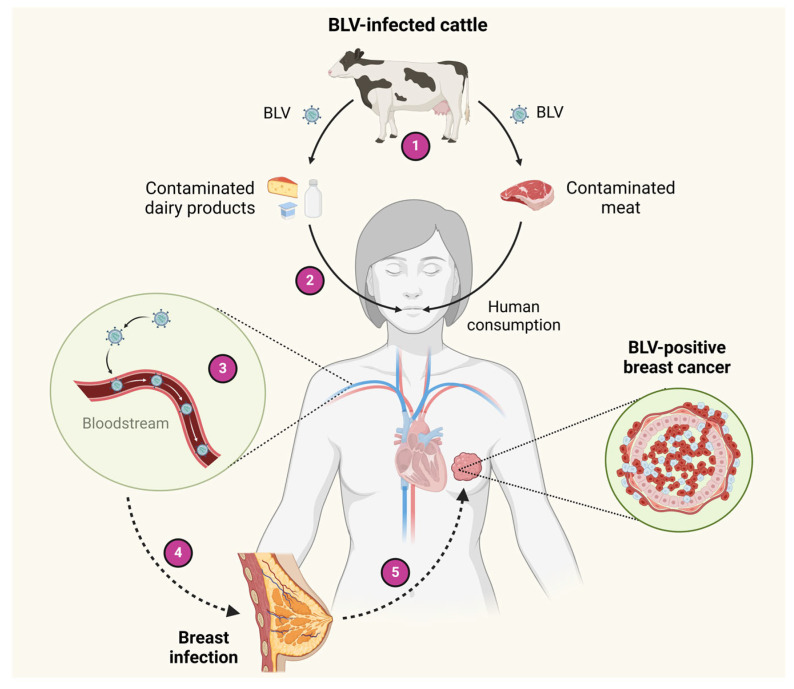
Hypothetical schemes of BLV transmission to humans. 1—Cattle can be infected with BLV. 2—BLV DNA can be transmitted from virus-infected cattle to milk and meat for human consumption. 3—After the ingestion of dairy products and meat infected by BLV, the virus can pass into blood circulation. 4—BLV can reach the breast tissue through blood circulation. 5—In the BLV-infected breast epithelial cells, the virus could cooperate with other factors to induce breast carcinogenesis. Created by BioRender.com.

A hypothetical scheme regarding the potential routes for BLV infection in human breast tissues through infected milk and meat consumption is shown in Figure 2. Other hypotheses for BLV entry to humans, such as direct contact with infected animals or through vaccine production processes that involve the use of BLV-contaminated fetal bovine sera, have also been proposed [88]. As occurs in cattle-to-cattle transmission, the lack of protection when handling infected animals, blood/tissue exposure, or inadequate safety protocols could theoretically be alternative routes for the transmission of BLV to humans [81,83]. Despite these potential risks, there are no confirmed reports of BLV transmitting infection in humans in this way. However, the detection of BLV in human blood cells may suggest a potential route of transmission to other individuals via the blood [30]. Further investigations are required to elucidate the routes by which BLV reaches human tissues.

## 4. BLV Entry into Human Cells

BLV shows a preferential tropism for B lymphocytes, but it was demonstrated that BLV in vivo also targets other leukocytes such as monocytes, granulocytes, and CD8+ T lymphocytes [89]. Moreover, BLV is able to infect a broad variety of cells, such as canine, feline, and murine cells, and also bovine mammary epithelial cells [27,28]. Furthermore, the capacity of BLV to infect a variety of human cells of different origins, such as embryonic kidney (293T) [90], cervical carcinoma (HeLa) [90], glioblastoma (U-118 MG), ovarian teratocarcinoma (PA-1) [91], and breast adenocarcinoma (MCF-7) [92], was demonstrated. Particularly, evidence suggests the tropism of BLV towards human breast epithelial cells, based on the fact that these epithelial cells express viral proteins after BLV infection [36,79,93]. Overall evidence suggests that the cellular receptors involved in BLV entry could be broadly expressed rather than restricted to a specific cell type.

It was reported that BLV is able to infect the human WI-38 cells (lung fibroblast cells) in vitro. However, WI-38 cells released only small amounts of the viral particles despite remaining infected, as measured by the expression of BLV antigen and the syncytia formation assay [94]. This assay evaluates the capacity of retroviruses to induce fusion between infected and uninfected cells, leading to the creation of multinucleated cells, known as syncytia. The syncytia formation assay is commonly used to monitor BLV infection in vitro [90,92]. Also, it demonstrated the capacity of BLV to infect human glioblastoma, neuroblastoma, and ovarian teratocarcinoma cells [91]. Interestingly, some human cell lines were susceptible to BLV infection, including MCF-102A (non-malignant) and MCF-7 (adenocarcinoma) breast cells. But, only MCF-7 cells were able to sustain BLV infection and also showed syncytia formation and multinucleated cells [92].

The high-affinity cationic amino acid transporter 1 (CAT1), also known as solute carrier family 7 member 1 (SLC7A1), is a transmembrane protein primarily responsible for the uptake of cationic amino acids into cells. This protein plays a critical role in cellular metabolism, growth, and immune response by regulating the availability of essential amino acids required for protein synthesis and other metabolic pathways [95]. SLC7A1 is considered a potential entry receptor for BLV into human epithelial cells. In fact, HeLa (human cervical carcinoma cells) and 293T (human embryonic kidney cells) expressing SLC7A1 formed syncytia in culture after inoculation with BLV [90]. Moreover, the knockdown of SLC7A1 using specific siRNAs significantly decreased both the binding between SLC7A1 and BLV Env protein and the BLV infection of CC81-GREMG cells [90]. In addition, overexpression of SLC7A1 was able to enhance the susceptibility of the same cells to BLV infection [96]. These facts together permit the consideration of the potential role of SLC7A1 in BLV binding and infection. Interestingly, the levels of SLC7A1 mRNA were significantly increased in BC tissues compared to normal breast samples (*p* < 0.01) [97].

**Figure 3:**
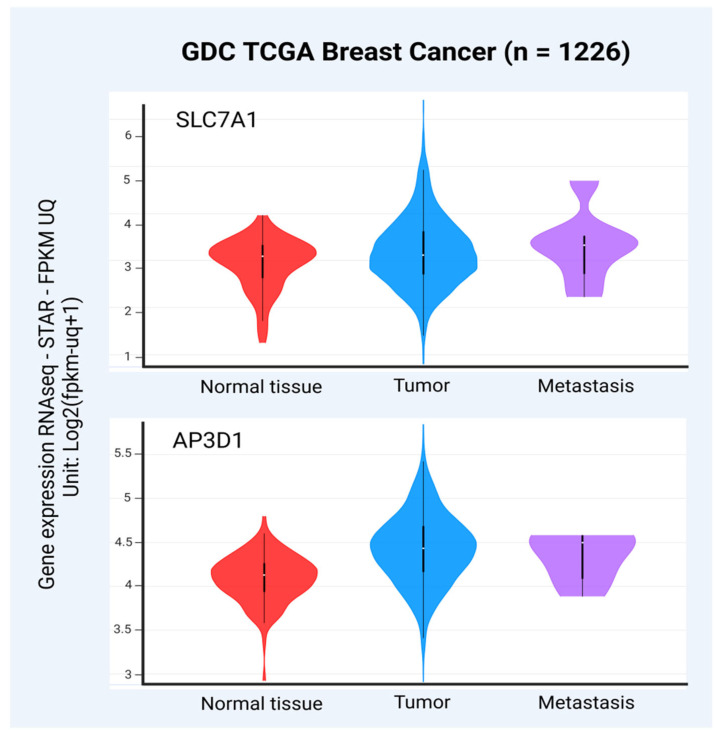
Levels of SLC7A1 and AP3D1 transcripts in primary BC, metastasis, and normal breast tissues (GDC TCGA BC, n = 1226 after removing samples with nulls). The levels of both SLC7A1 and AP3D1 mRNA were significantly increased in primary tumors and metastasis compared to normal breast samples (*p* = 0.0023 and *p* = 1.110 × 10^−16^, respectively, one-way ANOVA). The University of California, Santa Cruz (UCSC) genome database was used to obtain the raw data (https://xena.ucsc.edu). For the correlation of SLC7A1 and AP3D1 expression levels and type of samples, the UCSC Xena platform was used (https://xenabrowser.net accessed on 8 and 13 December 2024, respectively). The graphs were obtained from the Xena platform, and the figure was created using BioRender.com.

According to the TCGA database [98,99], SLC7A1 expression was also higher in BC tissues and metastasis compared to normal breast tissues (Figure 3). These facts together could suggest that increased levels of SLC7A1 make BC cells more susceptible to BLV infection (Figure 3). Moreover, it was demonstrated that the overexpression of SLC7A1 confers survival advantages and apoptosis resistance in MCF-7 and T47D BC cells [100].

The adaptor-related protein complex 3 subunit delta 1 (AP3D1) is a critical component of the adaptor protein complex-3 (AP-3), which is involved in intracellular protein trafficking [101]. This complex facilitates the sorting and transport of cargo proteins from the trans-Golgi network to lysosomes and other endosomal compartments [102]. The AP3D1 protein has also been proposed as a potential receptor for BLV in humans, sharing 88% of identity compared to the bovine receptor [103]. In fact, it was reported that HEp-2 human cancer cells ectopically expressing the bovine binding domain for BLV gp51 (clone BLVRcpl) had an increased susceptibility to BLV infection compared to untransfected control cells [104]. Furthermore, a significant increase in the sensitivity to BLV infection was evidenced in BLVRcpl-transfected cells after antibiotic selection, which highlights the potential role of this molecule in BLV infection [104]. Posteriorly, the BLV receptor (BLVR) is related to AP-3, which participates in intracellular protein transport [105]. According to The Cancer Genome Atlas (TCGA) database [98,99], AP3D1 expression was increased in primary BC and metastasis compared to normal breast tissues, suggesting again that BC cells with increased levels of AP3D1 could be more susceptible to BLV infection (Figure 3). Moreover, an increased AP3D1 gene expression was evidenced in invasive ductal carcinoma compared to invasive lobular carcinoma [106].

Taken together, these findings suggest that BLV entry into breast epithelial cells can be induced by overexpressing key receptors involved in this process. Nevertheless, further functional studies in breast epithelial cells are required to validate this hypothesis.

## 5. BLV Infection and the Risk of BC Development

Although the potential involvement of BLV in BC still remains controversial, the virus has been widely detected in BC samples and has been associated with an increased risk of tumor development [35,36,37]. For instance, a meta-analysis conducted by Khatami et al., which included nine case-control studies with a total of 826 BC cases and 898 individuals in the control groups, found an association between BLV infection and the risk of BC development (OR = 2.57; 95% CI: 1.45–4.56; *p* = 0.001) [107]. Another meta-analysis conducted by Saeedi-Moghaddam et al. including 11 studies, 3340 cases, and 635 controls also found an association of BLV infection and BC (OR = 3.92, 95% CI: 2.98–5.16; *p* < 0.00001) [108].

Particularly, BLV DNA was evidenced in 22/72 (30.5%) samples of BC tissues, which was statistically increased compared to samples from patients with healthy breast tissues (10/72; 13.9%) (OR = 2.73, 95% CI: 1.18–6.29; *p* = 0.027) [35]. Similarly, Olaya-Galán et al. found the occurrence of BLV DNA in 46/75 (61.3%) samples from BC cases and in 40/83 (48.2%) of the control tissues by nested PCR, linking the viral presence with an increased risk of BC development (OR = 2.45, 95% CI: 1.08–5.52; *p* = 0.031) [36]. While Buehring et al. (2015) demonstrated the presence of BLV DNA in 67/114 (59%) of BC samples using in situ PCR, and it was significantly increased compared to the 30/104 (29%) samples obtained from the controls (OR = 3.07, 95% CI: 1.66–5.69; *p* = 0.0004) [79].

Additionally, an increased presence of BLV DNA in premalignant breast tissues compared to healthy breast specimens was also demonstrated. In fact, Buehring et al. (2015) found an intermediate frequency of BLV DNA in premalignant breast tissues (8/21; 38%) between the BC and normal control groups (*p* for trend < 0.001) [79]. In the same way, Baltzell et al. (2018) obtained an increased frequency of BLV tax DNA by in situ PCR from normal breast specimens (20/103; 19.6%) compared to premalignant tissues (18/52; 34.0%) and BC plus ductal carcinoma in situ (DCIS) samples (49/89; 54.4%). In consequence, the presence of BLV *tax* DNA was related to an increased risk of BC or DCIS (OR = 5.25, 95% CI: 2.69–10.23; *p* < 0.0001) [37].

A study conducted by Lawson and Glenn (2017) analyzing the paired samples of benign breast tissue and subsequent BC specimens from the same patients revealed a higher frequency of BLV DNA in both benign breast tissues (18/23; 78.3%) and the later BC samples (20/22; 90.9%) compared to healthy breast specimens (6/17; 35.3%) [38]. Similarly, in a paired study, Buehring et al. (2017) observed an increased probability of BC development in women with BLV-positive results in both initial and subsequent samples, compared to those in whom BLV was either not detected or only positive in one of the paired specimens (*p* = 0.0484) [109].

Finally, Lendez et al. (2018) demonstrated that BLV DNA occurrence in BC tissues was associated with increased proliferation rates (*p* = 0.014) and HER-2 oncogene expression (*p* = 0.042) [110]. Similarly, Khan et al. reported a higher prevalence of BLV positivity in grade II invasive ductal carcinoma (IDC) (500/559; 89.4%) compared to grade I (59/559; 10.5%) and grade III tumors (0/559) [111]. However, other studies found no significant association between the presence of BLV and the tumor size, disease stage, estrogen receptor (ER) levels, progesterone receptor (PR) levels, human epidermal growth factor receptor 2 (HER-2) levels, proliferation index, or other clinicopathological features in BC patients [35,112,113]. Interestingly, it was suggested that the failure to efficiently eliminate BLV due to low binding affinity for HLA-II may contribute to BC development [114].

However, the presence of BLV DNA in BC tissues does not necessarily imply an active viral infection or causation. In this regard, the expression of the BLV p24 protein was detected in 10% of BC tissues [36], while Buehring et al. found the same protein in 12/236 (5.1%) of the BC specimens [79]. In addition, the presence of gp51 was evidenced in 7% of BC samples [93]. Furthermore, it was demonstrated that the MCF-7 cells were able to maintain a stable BLV infection over the 3-month follow-up, confirmed by both PCR and the immunohistochemical expression of the BLV p42 protein [92]. These facts together allow the consideration of a potential active replication of BLV in BC tissues.

Overall, the evidence indicates an increased frequency of BLV in BC tissues compared to non-tumor controls, suggesting a potential association between BLV infection and the risk of BC development. However, some studies have reported a decreased frequency of BLV infection in BC tissues [86,115,116], while others found no significant differences in BLV presence between BC cases and non-tumor controls [117]. The discrepancies regarding the potential association of BLV infection and BC may be attributed to differences in detection methods (PCR, in situ PCR, nested PCR) and also in the viral DNA sequence used as a target (*tax*, *gag*, *env*, LTR). Furthermore, differences in the amount of beef and dairy product consumption across the study populations (North American, South American, European, Australian, and Asian) could also explain the discrepancies between the frequencies of BLV DNA found in BC patients [86]. Additionally, it is important to consider that other risk factors, such as genetics and environmental exposure, may confound the observed association between BLV infection and BC development. Future studies should adjust for these variables to clarify the relationship. A summary of these studies is presented in Table 1.

## 6. Oncogenic Properties of the BLV Encoded Proteins

The oncogenic properties of BLV in animal and human cells have been demonstrated [54,120,121]. However, to the best of our knowledge, there is no information available regarding the direct role of BLV in human BC initiation or progression. This knowledge gap is critical given the potential implications of BLV as a zoonotic agent with oncogenic capabilities. This section focuses on the oncogenic potential of BLV, addressing three key areas: (1) BLV in bovine mammary cells, (2) the closely related HTVL-1 tax protein in human BC cells, and (3) BLV in human cells from different origins as potential models for a better understanding of BLV’s contribution to human BC.

BLV has been shown to enhance the proliferation rate of C72 bovine mammary cells, although these cells were unable to grow in soft agar, a hallmark of oncogenic transformation [120]. Infected bovine mammary epithelial cells (MAC-T) exhibited significantly higher expression levels of TNF-α mRNA compared to uninfected cells (*p* < 0.001) [122]. Conversely, another study reported reduced viability in BLV-infected MAC-T cells, accompanied by Bcl-2 downregulation and increased TLR9 mRNA expression [123]. Furthermore, the BLV Tax protein was observed to induce DNA damage and impair DNA repair mechanisms in mammalian cells, including bovine mammary epithelial cells [121]. BLV p34 was shown to cooperate with the Ha-ras oncogene in transforming rat embryo fibroblasts, which subsequently formed tumors when injected into nude mice [55].

On the other hand, some authors reported the potential role of the HTLV-1 tax protein in human BC development [124,125,126]. HTLV-1 is a closely related retrovirus to BLV that encodes a similar tax protein [127]. In this regard, the contribution of HTLV-1 tax protein to epithelial cell carcinogenesis could provide valuable information to elucidate the potential role of the BLV tax protein in BC development. For instance, it was reported that the HTLV-1 tax protein interacts with CREB-binding protein (CBP)/p300, inhibiting the activation of BRCA1 induced by ERα [124]. In addition, the HTLV-1 tax protein stimulated the E2–ERα-mediated expression of genes controlled by estrogen response elements (EREs) [125]. Moreover, the HTLV-1 tax protein has the capacity to inhibit BRCA1-mediated activation of p53 target promoters [126].

Finally, the capacity of BLV Tax to deregulate the expression of 122 genes (upregulated: 90, downregulated: 32) was demonstrated in HeLa cells ectopically expressing this molecule [54]. Among them, the overexpression of CYR61, FOS, JUN, RORA, NR4A2, RRAD, GEM, and TNFAIP6 was evidenced at both the transcript and protein levels. In contrast, BLV Tax induced the downregulation of ID2, TNFSF10, IFIT1, and IFIT3 [54]. The capacity of BLV Tax to induce the c-fos promoter activation in SW480 human carcinoma colon cells was demonstrated [128]. Interestingly, human glioblastoma cells with integrated BLV showed proliferation advantages compared to uninfected parental cells [91]. In non-small cell lung cancer (NSCLC), the presence of BLV was significantly associated with PSG4 and CPB2 downregulation [80]. Additionally, BLV Tax induced DNA damage and disrupted DNA repair mechanisms in H9 human T cells, consistent with observations in bovine mammary cells [121].

Collectively, these findings suggest that BLV may affect critical cellular functions, including DNA damage repair, cell proliferation, apoptosis resistance, and immune responses, thereby contributing to oncogenesis. The presence of BLV p24 and gp51 proteins in BC tissues, as detected by immunohistochemistry, supports the possibility of active viral infection [36,79,93]. However, despite the mounting evidence, the precise molecular mechanisms by which BLV proteins contribute to BC initiation and progression remain unclear. Therefore, further clinical and experimental studies are needed to establish the direct role of BLV proteins in BC initiation and progression. 

## 7. Conclusions

The present study showed evidence to support the correlation between BLV infection and BC development in women. Although the exact molecular mechanisms by which BLV could act as a cofactor in breast carcinogenesis are not fully understood, the present review highlights some possibilities. Firstly, the detection of BLV in dairy products and meat for human consumption jointly with the occurrence of anti-BLV antibodies in female blood makes possible the potential transmission of BLV to humans through the diet. Second, some host receptors involved in BLV attachment and fusion to epithelial cells are overexpressed in BC, which make BLV entry into human mammary epithelial cells plausible. Thirdly, the detection of BLV proteins in BC tissues, joined with the capacity of some viral proteins to disrupt key cellular pathways, makes the potential contribution of BLV to human breast carcinogenesis reasonable. However, further clinical and experimental studies focusing on the potential contribution of BLV to BC initiation and progression are strongly necessary. For instance, longitudinal studies following BLV-infected women would be valuable in assessing the long-term effects of BLV infection on BC development. Furthermore, additional experiments are necessary to evaluate the function of BLV-encoded proteins, including Tax, in human breast epithelial cells to enhance understanding of their possible role in BC development and progression. In the future, if BLV is confirmed as a risk factor for BC, it will be important to strengthen strategies to reduce exposure and potential viral transmission to humans, such as the expansion of screening programs to test for BLV presence in dairy products and beef.

## Figures and Tables

**Table 1:** Presence of BLV in BC tissues and controls.

Author, Year	Country	Tissue Type	Methods	Region/Gene	BLV Pos/Total (%)	Comments
Schwingel et al., 2019 [35]	Brazil	FFPE	Nested PCR	DNA	Control = 10/72 (13.9) BC = 22/72 (30.5)	No association of BLV with metastasis occurrence, grade of tumor differentiation.
Olaya-Galán et al., 2021 [36]	Colombia	FFPE	Nested PCR	*gag*, LTR, *tax*, and *env*	Control = 40/83 (48.2) BC = 46/75 (61.3)	OR = 2.45, 95% CI: 1.08–5.52; *p* = 0.031
Buehring et al., 2015 [79]	USA	FFPE	IS-PCR	*tax*	Control = 30/104 (29) Premalignant = 8/21 (38) BC = 67/114 (59)	OR = 3.07, 95% CI: 1.66–5.69; *p* = 0.0004 (control vs. cases)
Baltzell et al., 2018 [37]	USA	FFPE	IS-PCR	*tax*	Control = 20/103 (19.6) Premalignant = 18/52 (34.0) BC + DCIS = 49/89 (54.4)	OR = 5.25, 95% CI: 2.69–10.23; *p* < 0.0001 (control vs. cases)
Lawson and Glenn, 2017 [38]	Australia	FFPE	IS-PCR	*tax*	Control = 6/17 (35.3) Benign tissues = 18/23 (78.3) BC = 20/22 (90.9)	Benign breast tissues correspond to subsequent BC in the same patients.
Buehring et al., 2017 [109]	Australia	FFPE	IS-PCR	*tax*	Control = 19/46 (41.3) BC = 40/50 (80.0)	OR = 4.72, 95% CI: 1.71–13.05; *p* ≤ 0.003
Khalilian et al., 2019 [32]	Iran	FFPE	Nested PCR	*tax*	Control = 12/28 (42.9) BC = 48/172 (27.9)	-
				*gag*	Control = 4/28 (14.3) BC = 12/172 (7.0)	
Lendez et al., 2018 [110]	Argentina	FFPE	IS-PCR	*tax*	Nonmalignant = 1/4 (25.0) BC = 19/85 (22.6)	BLV was associated with increased proliferation rates and HER2 expression
Khan et al., 2022 [111]	Pakistan	FFPE	Nested PCR	*tax* and *gag*	Control * = 10/80 (12.5) BC = 728/2710 (26.8)	OR = 2.6, 95% CI: 1.35–5.19; *p* = 0.0029
Delarmelina et al., 2020 [113]	Brazil	FFPE	Nested PCR	*tax* and/or *env*	Control = 23/39 (59.0) BC = 47/49 (95.9)	No association of BLV with tumor size, HER2, Ki-67, ER, and PR.
Elmatbouly et al., 2023 [112]	Egypt	FFPE	Nested PCR	*tax*	Control = 2/50 (4.0) BC = 8/50 (16.0)	No association of BLV with some tumor features
Khasawneh et al., 2024 [118]	Jordan	FFPE	Nested PCR	*tax*	Control = 0/25 (0) BC = 19/103 (18.4)	*p* > 0.05 (control vs. cases) No association of BLV with some tumor features
Amato et al., 2023 [119]	USA	Fresh/Frozen	Nested PCR	*tax*	Control = 0/16 (0) BC = 0/30 (0)	-

Legend: FFPE, Formalin-fixed and paraffin-embedded tissues; IS-PCR, in situ PCR; BC, Breast cancer; DCIS, Ductal carcinoma in situ; * Breast diseases samples group including fibroadenoma, fibrocystic disease, granulamatous mastitis, phyllodes tumor, and invasive breast carcinoma.

## Abbreviations

The following abbreviations are used in this manuscript:

AP-3	Adaptor Protein Complex-3
AP3D1	Adaptor-related Protein Complex 3 Subunit Delta 1
ATM	Ataxia-Telangiectasia Mutated
BC	Breast Cancer
BLV	Bovine Leukemia Virus
BLVR	Bovine Leukemia Virus Receptor
BRCA1	Breast Cancer Gene 1
CAT1	High-affinity Cationic Amino acid Transporter 1
CBP	CREB-binding Protein
DCIS	Ductal Carcinoma in situ
DNA	Deoxyribonucleic Acid
EBL	Enzootic Bovine Leukosis
EBV	Epstein-Barr Virus
EMT	Epithelial-to-Mesenchymal Transition
ER	Estrogen Receptor
ERE	Estrogen Response Elements
FFPE	Formalin-Fixed and Paraffin-Embedded Tissues
gp	Glycoprotein
HCMV	Human Cytomegalovirus
HER-2	Human Epidermal Growth Factor Receptor 2
HMECs	Human Mammalian Epithelial Cells
HPV	Human Papillomavirus
HTLV-1	Human T-cell leukemia virus type 1
IDC	Invasive Ductal Carcinoma
INT	Integrase
IS-PCR	In situ Polymerase Chain Reaction
LTR	Long Terminal Repeat
miRNA	Micro Ribonucleic Acid
MMTV	Mouse Mammary Tumor Virus
NSCLC	Non-Small Cell Lung Cancer
OR	Odds Ratio
PCR	Polymerase Chain Reaction
PR	Progesterone Receptor
RNA	Ribonucleic Acid
RNAPII	RNA Polymerase II
RT	Reverse transcriptase
SLC7A1	Solute Carrier Family 7 Member 1
TCGA	The Cancer Genome Atlas
TLR9	Toll-Like Receptor 9
TNF-α	Tumor Necrosis Factor Alpha
UCSC	University of California, Santa Cruz
